# Molecular Regulation of *Trypanosoma congolense*-Induced Nitric Oxide Production in Macrophages

**DOI:** 10.1371/journal.pone.0059631

**Published:** 2013-03-25

**Authors:** Rani Singh, Bruce C. Kone, Abdelilah S. Gounni, Jude E. Uzonna

**Affiliations:** 1 Department of Immunology, Faculty of Medicine, University of Manitoba, Winnipeg, Manitoba, Canada; 2 University of Texas Medical School, Houston, Texas, United States of America; Institut Jacques Monod, France

## Abstract

BALB/c mice are highly susceptible while C57BL/6 mice are relatively resistant to experimental *Trypanosoma congolense* infection. Several reports show that an early interferon-gamma (IFN-γ) response in infected mice is critically important for resistance via the activation of macrophages and production of nitric oxide (NO). NO is a pivotal effector molecule and possesses both cytostatic and cytolytic properties for the parasite. However, the molecular mechanisms leading to *T. congolense* (TC)-induced NO release from macrophages are not known. In this study, we investigated the signaling pathways induced by trypanosomes in immortalized macrophage cell lines from the highly susceptible BALB/c (BALB.BM) and relatively resistant C57Bl/6 (ANA-1) mice. We found that *T. congolense* whole cell extract (TC-WCE) induces significantly higher levels of NO production in IFN-γ-primed ANA-1 than BALB.BM cells, which was further confirmed in primary bone marrow-derived macrophage (BMDM) cultures. NO production was dependent on mitogen-activated protein kinase (MAPK, including p38, Erk1/2, and JNK) phosphorylation and was significantly inhibited by specific MAPK inhibitors in BALB.BM, but not in ANA-1 cells. In addition, *T. congolense-* and IFN-γ-induced NO production in ANA-1 and BALB.BM cells was dependent on STAT1 phosphorylation and was totally suppressed by the use of fludarabine (a specific STAT1 inhibitor). We further show that *T. congolense* induces differential iNOS transcriptional promoter activation in IFN-γ-primed cells, which is dependent on the activation of both GAS1 and GAS2 transcription factors in BALB.BM but only on GAS1 in ANA-1 cells. Taken together, our findings show the existence of differential signalling events that lead to NO production in macrophages from the highly susceptible and relatively resistant mice following treatment with IFN-γ and *T. congolense*. Understanding these pathways may help identify immunomodulatory mechanisms that regulate the outcome of infection during Trypanosome infections.

## Introduction

African trypanosomiasis, also known as sleeping sickness, is a parasitic disease of humans and livestock that is transmitted by various species of tsetse fly belonging to the genus *Glossina. Trypanosoma congolense*, *T. vivax* and *T. brucei brucei* are the major cause of disease in livestock [1]. The disease causes significant mortality in both humans and livestock and significantly impacts on economic development of sub-Saharan African countries where it is endemic. It is estimated that direct losses attributed to African trypanosomiasis exceed US$ 4.75 billion/year [2]. In addition, the indirect effect on public health is also enormous, as infected animals can serve as a reservoir for the tsetse transmission to human [3], [4].

Trypanotolerance, or the capacity of some indigenous West African cattle breeds such as the N'dama to remain productive despite being infected, is correlated with a genetic capacity to limit parasitemia, anaemia and production of proinflammatory cytokines [5]. In order to investigate the disease pathogenesis and to test new drug therapies, small animal models have been used. Uniquely, certain aspects of the disease in these animal models moderately mimic the disease in cattle. For instance, C57Bl/6 mice are considered relatively resistant because they can control several waves of parasitemia and survive up to 80–120 days after infection [6]. In contrast, the BALB/c mice are highly susceptible and succumb within 8–10 days post-infection without controlling the first wave of parasitemia [6]. Macrophages are professional antigen-presenting cells (APCs) that act as first line of defense against pathogens via phagocytosis and release of proinflammatory cytokines, [7]. Importantly, macrophages play a critical role in the control of many protozoan parasitic infections including African trypanosomiasis. The parasiticidal activities of macrophages has been shown to correlate with changes in their inducible nitric oxide synthase (iNOS) gene expression and nitric oxide (NO) production [7], [8]; which is in part related to the levels of interferon-gamma (IFN-γ) production by T cells.

We previously showed that *Trypanosoma congolense* (TC) induces differential production of NO in macrophages from the highly susceptible BALB/c and relatively resistant C57Bl/6 mice [9]. However, the molecular mechanisms leading to TC-induced NO release from macrophages are completely unknown. Emerging evidence suggest that both mitogen-activated protein kinases (MAPKs) and signal transducer and activator of transcription (STAT) family members can coordinately interact to propagate multiple intracellular signalling cascades that lead to pro-inflammatory cytokine responses and NO production. Thus, MAPKs and their upstream family kinase members activate a number of transcription factors and induce transcription of a plethora of inflammatory genes in response to microbial products and cytokines [10]. Additionally, MAPKs are involved in responses to an array of extracellular stimuli such as mitogens, growth factors, pathogen products, and other physical stress factors [11].

In this report, we investigated the differential signaling events leading to NO production in TC whole cell extract-treated macrophage cell lines from the relatively resistant and highly susceptible mice in the presence or absence of IFN-γ treatment. Collectively, our findings show that the signalling events that lead to NO production are differentially regulated in macrophages from the highly susceptible and relatively resistant mice following treatment with IFN-γ and *T. congolense.*

## Materials and Methods

### Ethics Statement

All mouse experiments were approved by the University of Manitoba Animal Care Committee in accordance with the regulation of the Canadian Council on Animal Care.

### Reagents

Recombinant mouse IFN-γ was purchased from Peprotech, Inc. (Rocky Hill, NJ). LPS from E. coli was purchased from DIFCO Laboratories (Detroit, MI, USA). Rabbit anti-mouse p38 MAPK mAb, rabbit anti-mouse ERK1/2 mAb, affinity-purified rabbit anti–phospho-p38 MAPK (Thr180/Tyr182), affinity-purified mouse anti–phospho-ERK1/2 (Thr202/Tyr204), rabbit anti-total and phospho-specific SAPK/JNK (Thr183/Tyr185) Abs, rabbit polyclonal anti-STAT1, and anti–phospho-tyrosine–specific STAT1 (#9172) mAbs were purchased from Cell Signaling Technology (Danvers, MA). All cell culture media (DMEM and RPMI), antibiotics (penicillin/streptomycin), and cell culture reagents were procured from Invitrogen Canada (Burlington, Ontario, Canada). FBS was purchased from HyClone Laboratories (Logan, UT). The p38 MAPK inhibitor 4- (4-fluorophenyl)-2-(4-methyl-sulfinylphenyl)-5-(49-pyridyl)-[1H]-imidazole (SB-203580), JNK inhibitor anthra (1,9-cd)pyrazol-6(2H)-one; 1,9-pyrazoloanthrone (SP-600125), and p42/p44 ERK inhibitor 1,4-diamino-2,3-dicyano-1,4-bis (2-aminophenylthio) butadiene (U-0126) were purchased from Calbiochem (Mississauga, Ontario, Canada). Fludarabine (specific inhibitor of STAT-1) was obtained from Sigma-Aldrich (Mississauga, Ontario, Canada). All other reagents were from Sigma-Aldrich unless stated otherwise.

### Experimental Animals

Six to eight week old female C57Bl/6 and BALB/c mice were purchased either from Charles River Laboratory, St. Constante, Quebec or from the University of Manitoba Central Animal Care Services (CACS) breeding facility. Female Swiss white CD1 mice, 5–6 wk old were also purchased from University of Manitoba (CACS) for expanding the trypanosome stabilates *in vivo*. All mice were housed in a specific pathogen free environment at the CACS and were maintained according to the recommendations of the Canadian Council of Animal Care.

### Culture of Immortalized Cell Lines and Primary Bone Marrow Derived Macrophages (BMDM)

Two types of murine macrophage cell lines were used in this study. The origins of retrovirus-immortalized bone marrow-derived macrophage cell lines from relatively resistant C57Bl/6 (ANA-1 cells) and highly susceptible BALB/c (BALB.BM cells) mice used in this study have been previously described [12], [13]. BALB.BM and ANA-1 cells were cultured in complete RPMI-10 medium (RPMI medium supplemented with 10% heat inactivated fetal bovine serum [FBS], 2 mM L-glutamine, 100 U/ml of penicillin and 100 µg/ml of streptomycin). Primary bone marrow-derived macrophages (BMDMs) from BALB/c and C57BL/6 mice were generated as previously described by others [14], [15] and were cultured in complete RPMI-10 medium. Cells were maintained at 37°C in a humidified incubator containing 5% CO_2_.

### Trypanosomes and Preparations of Trypanosomal Whole Cell Extract

*T. congolense*, Trans Mara strain, variant antigenic type (VAT) TC13 was used in this study [16]. Frozen stabilates of *T. congolense* were used to infect immunosuppressed CD1 mice and were passaged every third day as described by others [16], [17]. The parasites were then purified from the infected mice by DEAE-cellulose chromatography [16], [17]. Parasites isolated by DEAE-cellulose were washed and resuspended in PBS at a final concentration of 10^8^/ml. Trypanosomes were mechanically disrupted by sonication and freeze/thawing several times, aliquoted and stored at −80°C until used. The endotoxin level in the preparation was <0.005 EU.

### Western Blot to Assess Phosphorylation of MAPKs and STATs

ANA-1 and BALB.BM cells were grown in RPMI-10 in Petri dishes and after they reached 90–95% confluency, were synchronized for 24 hr in serum free medium. Thereafter, the cells were treated with recombinant murine IFN-γ (500 IU/ml) alone, *T. congolense* whole cell lysate (TC-WCE, 1∶10 ratio) alone, or both IFN-g and WCE for 30, 60, 120 min. At selected time intervals, the cells were washed with ice-cold PBS, and total protein was extracted using M-PER lysis buffer (Thermo Fisher Scientific, ON, Canada) supplemented with a protease inhibitor cocktail, 1 mM sodium orthovanadate and 1 mM phenylmethylsulfonyl fluoride (Sigma, Mississauga ON, Canada). The cell lysates were centrifuged for 10 min at 4°C to pellet the cellular debris and the supernatants were collected and stored at −80°C for further use.

For western blot, the lysates (10 µg) were resolved in 10% SDS-PAGE, transferred unto polyvinylidene difluoride (PVDF) membranes (Amersham Biosciences, Baie D’Urfe, Quebec, Canada) and blocked with 5% BSA in TBST for 2 h at room temperature. Thereafter, the membranes were incubated at 4°C overnight with specific polyclonal rabbit antibodies against phosphorylated p38, JNK, ERK1/2, and STAT1. The membranes were further washed 5 times with TBST (5 min per wash) and probed with goat anti-rabbit HRP conjugated secondary Abs. Specific bands corresponding to phosphorylated molecules of interest were detected with ECL Advanced reagents (Amersham Biosciences). The blots were then stripped routinely and reprobed with antibodies against total p38, ERK1/2, JNK, STAT1 and beta-Actin (used as respective loading controls). Densitometric analysis was performed on developed blots as previously described [18] and data are presented as fold increase in phosphorylated compared to total over time zero.

### Griess Reaction

Nitrite concentration in the culture supernatants was measured by using Griess reagent as previously described [19]. Briefly, ANA-1 and BALB.BM cell monolayers were stimulated with TC, IFN-γ (500 IU/ml), or both and at indicated times, the supernatants were centrifuged at 1200 rpm for 10 min to remove cellular debris and the concentration of nitrite in the supernatant fluids was determined. In some experiments, cells were also incubated with SB203580 (50 µM), SP600125 (10 nM), and U0126 (50 µM) for 1 hr or with Fludarabine (50 µM) for 2 h prior to treatments with TC or IFN-γ. The inhibitors were purchased from Calbiochem (Mississauga, Ontario, Canada).

### Luciferase Reporter Constructs and Cell Transfection

ANA-1 and BALB.BM cells were seeded into 12-well culture plates in fresh complete medium and when they reached 70–90% confluency, the cells were transiently transfected with plasmid constructs containing wild-type (WT) promoters for mouse iNOS gene, or plasmid constructs containing mutations in transcription factor binding sites for interferon gamma activated site (GAS)-1 (GAS1Δ), GAS2 (GAS2Δ), or GAS1 and 2 (GAS1-2Δ). Transient transfection was performed using Turbofect™ *in vitro* transfection reagent (Fermentas Life Sciences, ON, Canada) according to the manufacturer’s suggested protocols. After 24 hr, the medium was changed and the transfected cells were washed twice with fresh medium and stimulated with IFN-γ (500 IU/ml), *T. congolense* WCE or both. The luciferase activity was measured by the Dual-Luciferase Assay System kit (Promega, Madison, WI) by using a luminometer (Berthold Bad Wildbad, Germany).

### Statistical Analysis

Data represents mean±SEM of experiments performed at least in duplicate. Statistical significance was determined by using unpaired Student’s *t* test by using GraphPad Prism software version 4.0. The P values <0.05 were considered statistically significant.

## Results

### *T. congolense* (TC) WCE Differentially Affects IFN-γ-induced NO Release in Macrophages from Resistant and Highly Susceptible Mice

**Figure 1:**
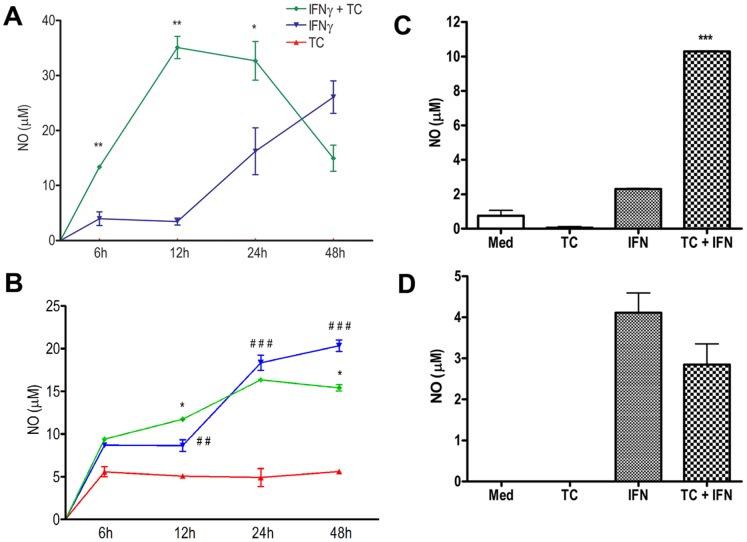
*T. congolense* augments IFN-γ-induced NO release in immortalized and primary bone-marrow derived macrophages from relatively resistant C57BL/6 mice. Macrophage cell lines from (A) relatively resistant (ANA-1) and (B) highly susceptible (BALB.BM) mice were serum-deprived for 24 h followed by stimulation with IFN-γ and TC WCE in combination or alone at indicated times. In some experiments, primary BMDM from (C) relatively resistant C57BL/6 and (D) highly susceptible BALB/c mice were similarly stimulation for 24 h. Supernatants were collected, centrifuged to remove cellular debris, and NO release was measured by Greiss’ Reaction. Data presented are representative of 5 different experiments with similar findings. *, p<0.05; **, p<0.01; and ***, p<0.001 compared to respective IFN-γ-stimulated controls; ##, p<0.01; ###, p<0.001 TC WCE+IFNγ compared to TC WCE.

Earlier studies have shown that NO plays a key role in orchestrating inflammatory cytokine gene expression and killing of pathogenic parasites including *T. congolense* [8], [9], [20]. In particular, NO has been shown to have both cytostatic and cytolytic effects against African Trypanosomes [9], [21], [22], and iNOS deficient mice are highly susceptible to *T. congolense* infection [23]. Because previous reports show that priming of macrophages with IFN-γ enhances NO production in parasite-infected macrophages [24], [25], we first investigated whether IFN-γ also enhances NO release in macrophage following treatment with WCE. Our results show that IFN-γ-primed and WCE-treated ANA-1 cells produced significantly higher NO (Fig. 1A) at 6, 12, and 24 h (p = 0.0086, p = 0.0022 and p = 0.04, respectively; n = 5) than similarly treated BALB.BM cells (Fig. 1B). Similar to immortalized cell lines, IFN-γ-primed and WCE-treated primary BMDMs from the relatively resistant C57BL/6 mice produced significantly (p<0.001, n = 3) more NO than similarly-treated cells from the highly susceptible BALB/c mice (Fig. 1C and D), suggesting that the effects observed in cell lines are real and not related to the immortalization process. Interestingly, while IFN-γ and WCE co-treatment upregulates NO production in both immortalized and primary macrophages from C57BL/6 mice (Fig. 1A and C), WCE co-treatment appears to either have no effect or suppress the effect of IFN-γ alone on macrophages from BABL/c mice (Fig. 1B and D). Moreover, whereas WCE alone induced modest level of NO release in BALB.BM cells; it did not have any effect in ANA-1 cells (Fig. 1A and B). Collectively, these results indicate that WCE differentially influence IFN-γ-induced NO release in macrophages from the relatively resistant (C57Bl/6) and highly susceptible (BALB/c.

### *T. congolense* WCE Mediates Differential Phosphorylation of MAPKs in BALB.BM and ANA-1 Macrophage Cell Lines

**Figure 2:**
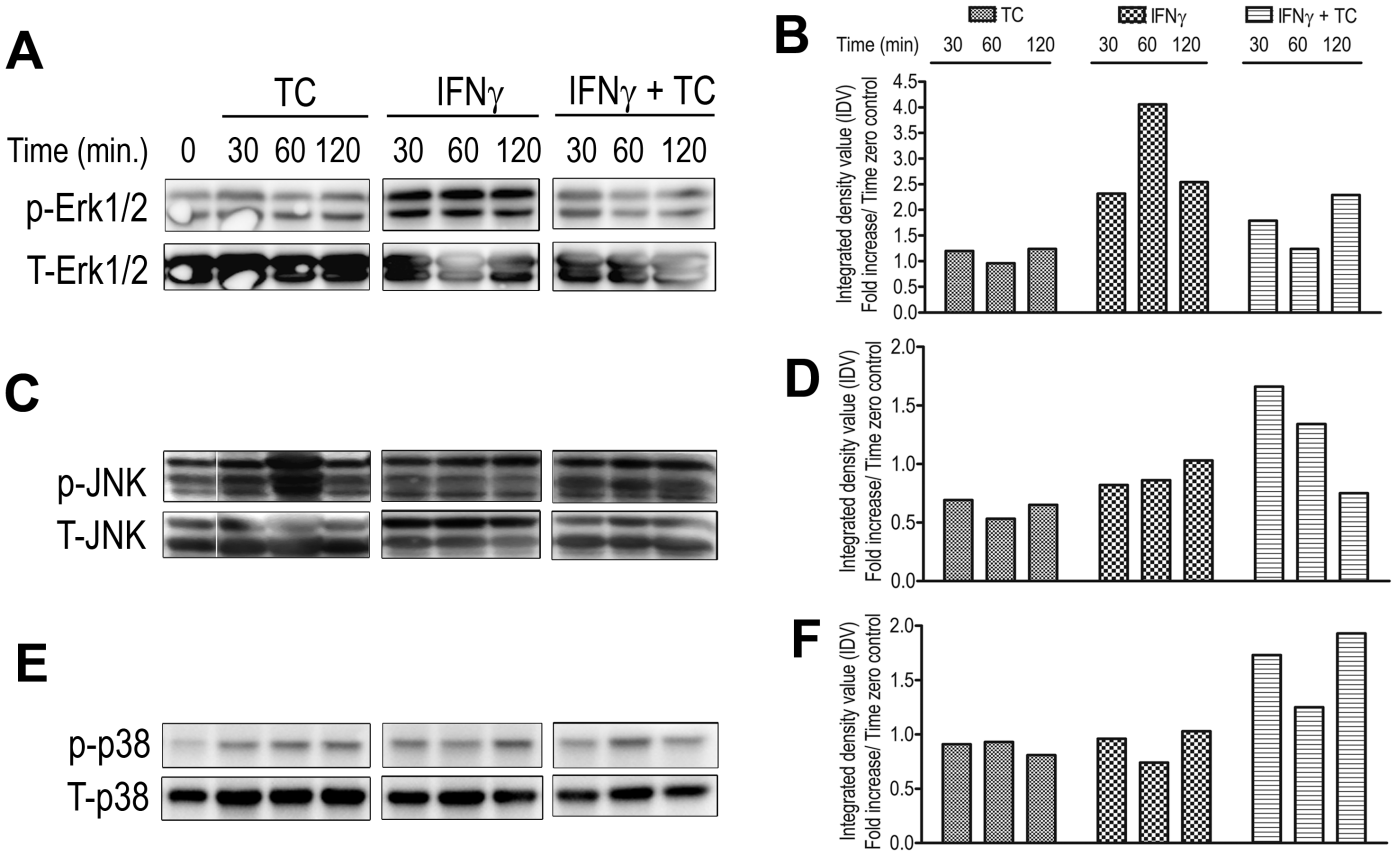
TC-WCE modulates IFNγ-induced MAPK Phosphorylation in ANA-1 cells. Macrophage cells (ANA-1) from relatively resistant C57BL/6 mice were serum-deprived for 24 h and stimulated with IFN-γ and TC in combination or alone. Cells were washed, and cell lysates were collected at indicated times. Erk1/2, JNK, and p38 MAPK phosphorylation (A, C, E) was assessed by Western blotting. The membranes were then stripped and re-probed for total (T) MAPK. Phosphorylation intensity was quantified by densitometry and presented in panels beside the blots as fold-increase in phosphorylation normalized with total MAPKs over time zero (B, D, F). Data presented are representative of 3 different experiments with similar findings.

**Figure 3:**
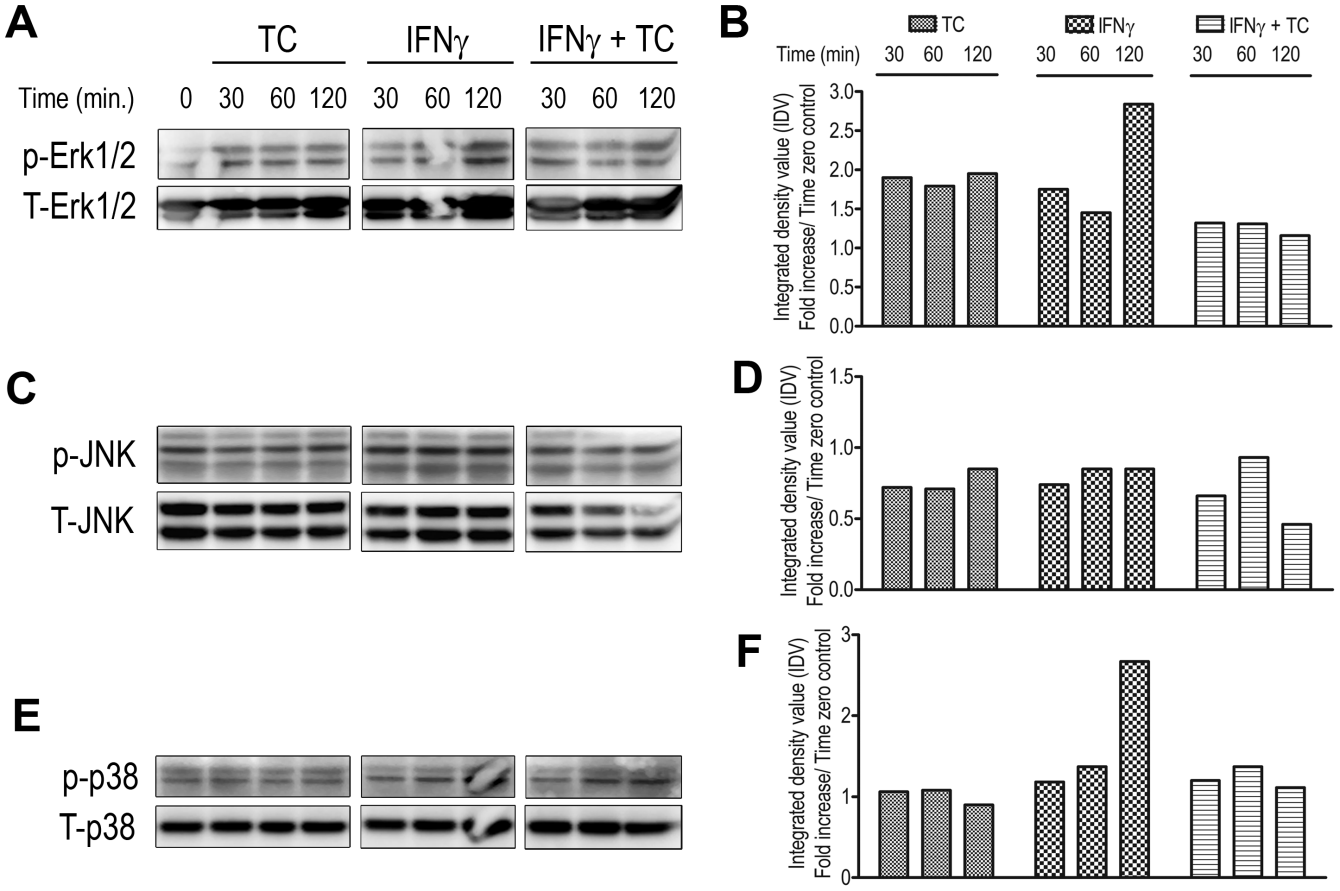
TC-WCE modulates IFN-γ-induced MAPK Phosphorylation in BALB.BM cells. Serum-deprived macrophage cell line BALB.BM from highly susceptible mice were stimulated with IFN-γ and TC in combination or alone, and cell lysates were collected at indicated times. Erk1/2, JNK, and p38 MAPK phosphorylation (A, C, E) was assessed by Western blotting. The membranes were stripped and re-probed with total MAPK antibodies for loading control. Phosphorylation intensity was quantified by densitometry and presented in panels beside the blots as fold-increase in phosphorylation normalized with total MAPKs over time zero (B, D, F). Data presented are representative of 3 different experiments with similar findings.

Next, we sought to investigate the underlying mechanisms that regulate the differential release of NO by IFN-γ-primed and WCE-treated macrophages from resistant and susceptible mice. MAPK signaling pathways have been identified as the upstream kinases that induce the activation of transcriptional pathways that lead to NO production [26]. To assess whether MAPKs contribute to the differential NO expression, we performed western blot analysis on cell lysates from IFN-γ-primed, WCE-treated macrophages using specific Abs against the phosphorylated regulatory sites on MAPKs. A slight increase in phosphorylation of ERK1/2 was observed in treated ANA-1 cells at 120 min (Fig. 2A and B) whereas ERK1/2 phosphorylation in BALB.BM cells was suppressed or not increased above the baseline (Fig. 3A and B). Similarly, IFN-γ and WCE induced JNK phosphorylation in ANA-1 cells at 30 to 120 min (Fig. 2C, D) post treatment whereas similarly treated BALB.BM cells showed no or below basal level phosphorylation (Fig. 3C, D). WCE induced a significant and sustained increase in p38 phosphorylation in ANA-1 cells starting at 10 min and lasting up to 120 min (Fig. 2E and F). In contrast, the phosphorylation of p38 in BALB.BM cells was only evident at 60 min post-treatment and declined thereafter such that by 120 min, p38 phosphorylation was significantly lower than the baseline (untreated cells, Fig. 3E and F). Collectively, these results show that treatment with TC and IFN-γ induces differential activation of MAPK in ANA-1 and BALB.BM macrophages.

### Inhibitors of ERK1/2, JNK, and p38 abolish TC and IFN-γ-induced NO Generation in ANA-1 and BALB.BM Cells

**Figure 4:**
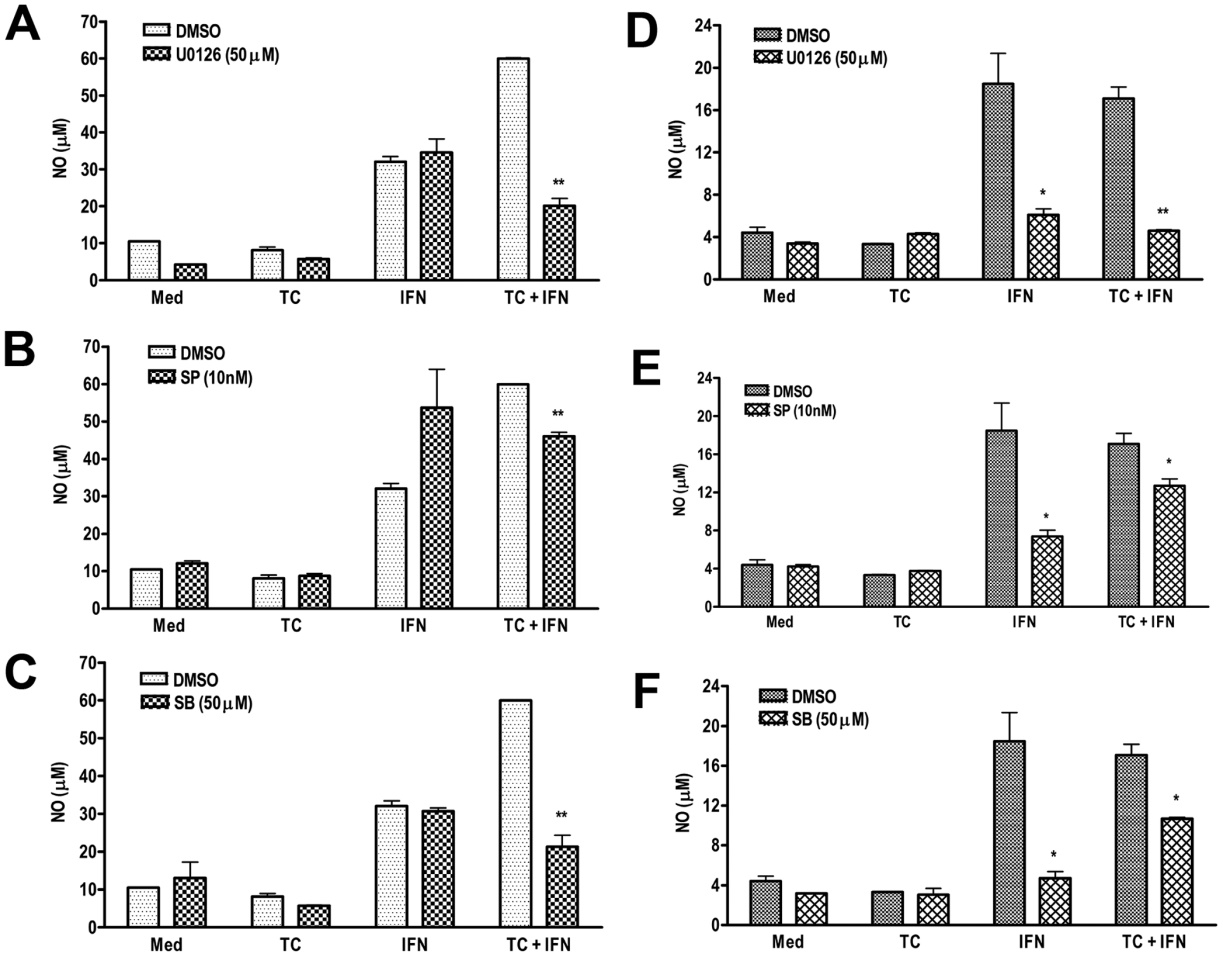
MAPK (Erk ½, JNK, and p38) inhibitors abrogate the TC and IFN-γ-induced NO release in macrophage cell lines. Serum-deprived ANA-1 (A, B, C) and BALB.BM (D, E, F) were incubated with MAPK inhibitors (U0126 for Erk1/2, A and D; SP600125 for JNK, B and E; and SB203580 for p38, C and F) for 1 h prior to stimulation with IFN-γ and TC in combination or alone, and nitric oxide (NO) release was measured in supernatants by Greiss’ Reaction. Data presented are representative of 3 different experiments with similar findings. *, p<0.05; **, p<0.0 1 compared to respective stimulated cells treated with DMSO (vehicle).

To further confirm the involvement of MAPKs in *T. congolense*/IFN-γ-induced NO release, we performed experiments using specific inhibitors of p38 (SB203580), p42/p44 ERK (U0126) and JNK (SP600125) MAPKs. An optimal dose of these inhibitors was first determined by assessing the NO inhibitory effect without any cytotoxicity (data not shown). Pre-treatment of ANA-1 cells with SB203580, U0126 and SP600125 significantly inhibited the release of NO following stimulation with IFN-γ and WCE (Fig. 4A–C). Similarly, pre-treatment of BALB.BM cells with U0126, SB203580 or SP600125 before stimulation with IFN-γ either alone or in combination with *T. congolense* caused a significant (p<0.05) inhibition of NO release (Fig. 4D–F), although the effects were more pronounced than in ANA-1 cells. Whereas the effect of MAPK inhibitors was largely inconspicuous on IFN-γ-induced NO production in ANA-1 cells (Fig. 4A–C), they completely abrogated the IFN-γ-induced NO release in BALB.BM cells (Fig. 4D–F). Collectively, these results shows that the key members of MAPK (JNK, Erk and p38) play a role in controlling intracellular signalling events that lead to the production of NO in IFN-γ/*T. congolense*-treated macrophages from both the highly susceptible and relatively resistant mice.

### STAT1 Regulates TC-induced NO Release in ANA-1 and BALB.BM Cells

**Figure 5:**
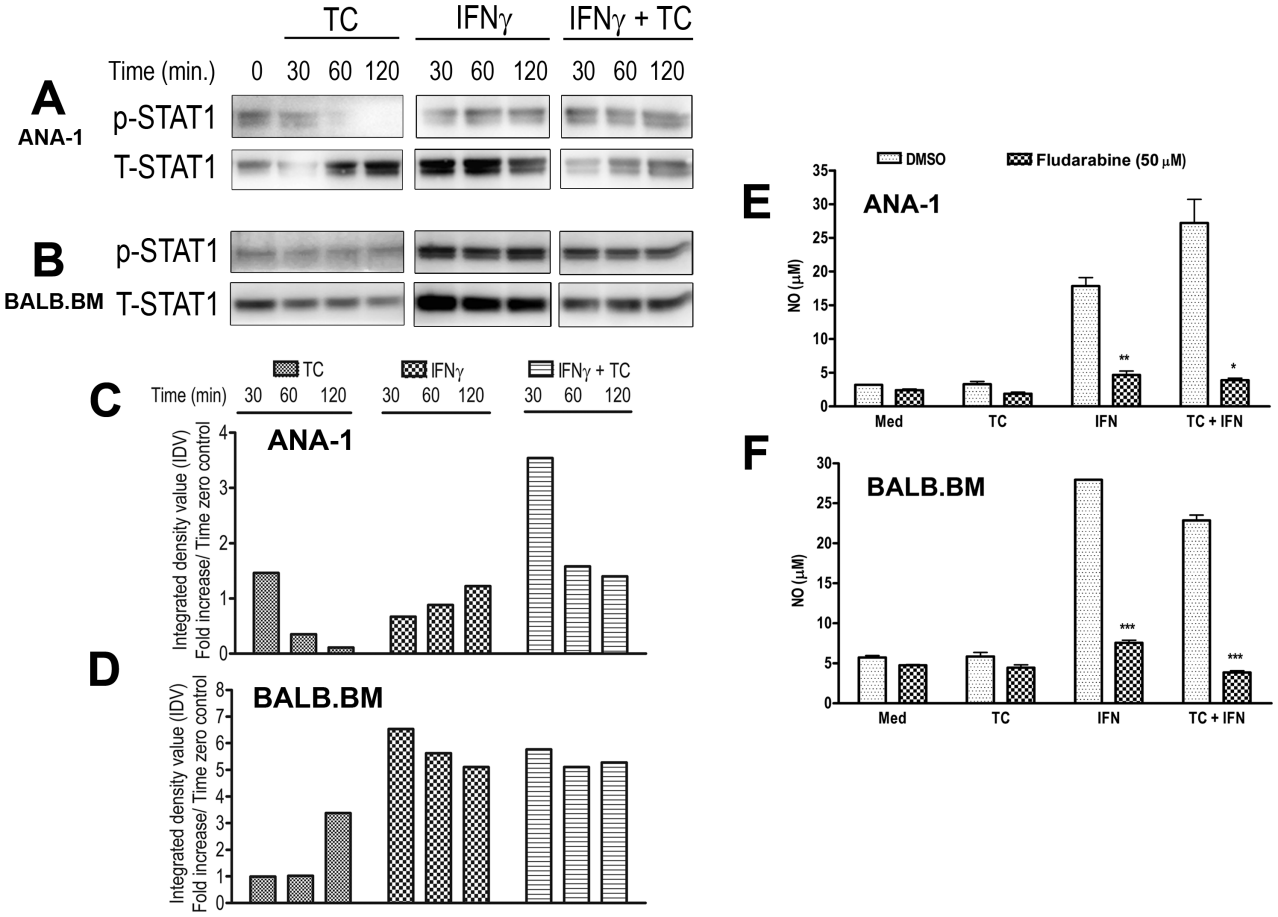
STAT1 regulates *T. congolense* WCE and IFN-γ-induced NO release in both ANA-1 and BALB.BM cells. Serum-deprived ANA-1 (**A**, **C**, **E**) and BALB.BM (**B**, **D**, **F**) macrophage cell lines were stimulated with TC, IFN-γ or both to assess STAT1 phosphorylation (**A**, **B**), also shown quantitatively in **C**, and **D**. In parallel, some cells were incubated with 50 µM STAT1 inhibitor (Fludarabine) prior to stimulation with IFN-γ and TC in combination or alone; and nitric oxide (NO) release was measured in supernatants by Greiss’ assay. Data presented are representative of 3 different experiments with similar findings. *, p<0.05; **, p<0.01; ***, p<0.001 compared to respective stimulated cells with DMSO (vehicle).

JAK-STAT signaling cascade is one of the core pathways that regulate responsiveness of macrophages to IFN-γ [7]. A previous study has shown that TLR-9-dependent recognition of *Trypanosoma brucei* soluble variant surface glycoprotein (sVSG) containing glycosylinositolphosphate (GIP-sVSG) by macrophages leads to STAT1 phosphorylation [7]. Moreover, cells from STAT1 deficient (STAT1^−/−^) mice do not respond to IFN-γ stimulation leading to enhanced susceptibility to bacterial and viral infections [27], [28]. To investigate the role of STATs signaling in *T. congolense*-induced NO production, we performed western blot analysis on macrophage lysates from both the highly susceptible (BALB.M) and relatively resistant (ANA-1) mice following stimulation with IFN-γ, TC or both. We did not observe any significant effect on STAT3 and STAT5 phosphorylation (data not shown). In contrast, STAT1 tyrosine phosphorylation was very evident, peaking in *T. congolense* and IFN-γ-stimulated ANA-1 cells peaking at 30 min and declining after 60–120 min (Fig. 5A, C). Interestingly, STAT1 phosphorylation following *T. congolense* and IFN-γ stimulation was sustained in BALB.BM cells (Fig. 5B, D).

To confirm the role of STAT1 in TC and IFN-γ-induced NO release, we treated ANA-1 and BALB.BM cells with fludarabine (a specific inhibitor of STAT1) prior to stimulation with *T. congolense* and IFN-γ. Treatment of ANA-1 and BALB.BM cells with fludarabine led to a significant inhibition in IFN-γ and *T. congolense*-induced NO release (Fig. 5E and F). Collectively these observations suggest a significant role of STAT1 signaling in *T. congolense* and IFN-γ-induced NO release macrophages.

### *T. congolense* WCE Induces NO Production through Activation of iNOS GAS1 and GAS2 Elements in Murine Macrophages

**Figure 6:**
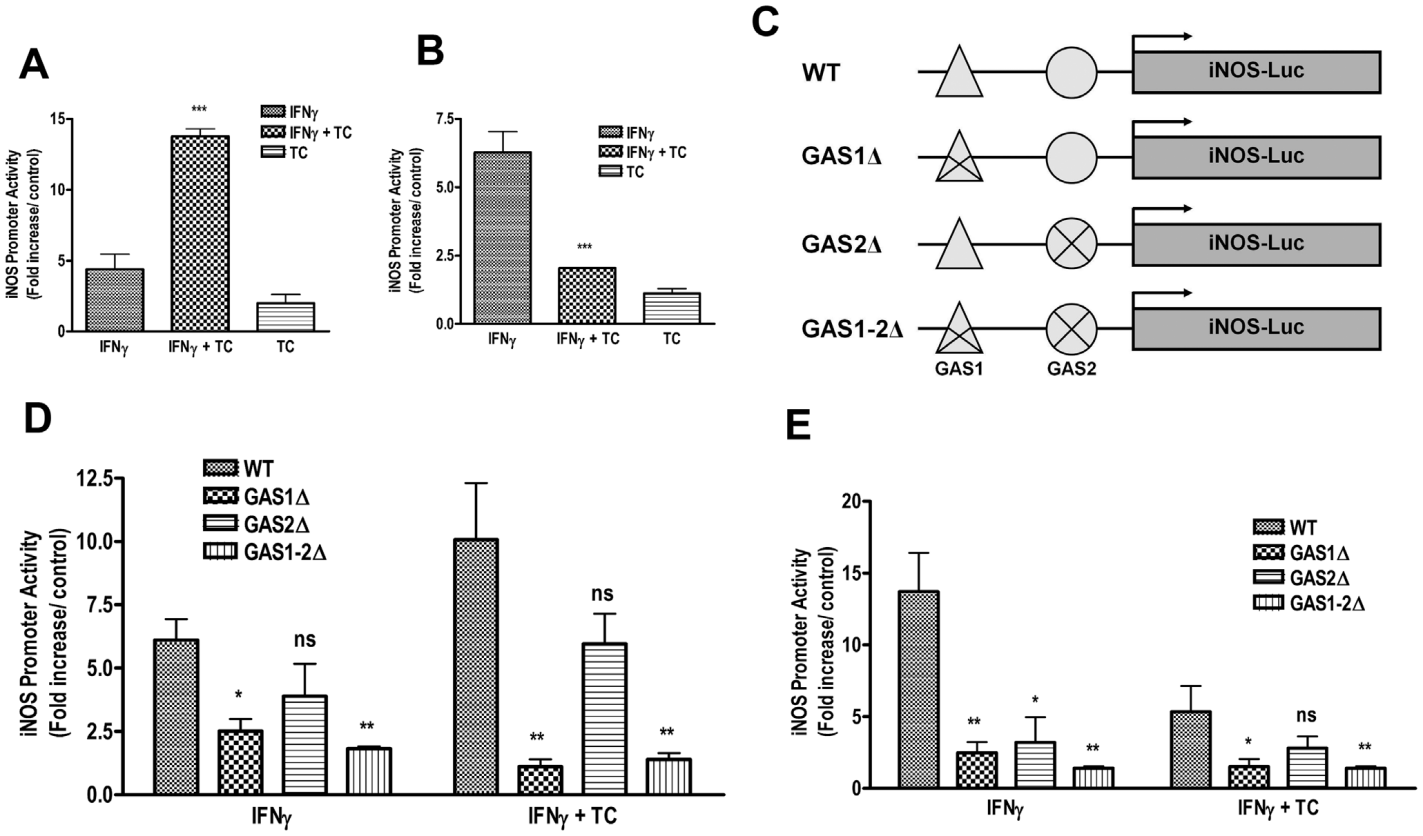
*T. congolense* and IFN-γ-induced iNOS promoter activity is regulated by GAS1 in ANA-1, and GAS1 and GAS2 transcription factors in BALB.BM. Serum-deprived macrophage cell lines ANA-1 (A, D) and BALB.BM (B, E) were transiently transfected with wild-type (WT) luciferase construct harboring iNOS promoter sequence. Transfected cells were stimulated with *T. congolense* WCE, IFN-γ or both to assess iNOS promoter activity (A, B). Additionally, cells were transfected with constructs containing WT or mutated at GAS1, GAS2, or both (D, E); a schematic of mutants is shown in C. Cells were then stimulated with IFN-γ, TC or both; and iNOS promoter luciferase activity was measured by dual-luciferase assay kit (Promega). Data represent fold increase in (Renilla) normalized iNOS promoter activity compared to unstimulated control and are representative of 3 different experiments with similar findings. *, p<0.05; **, p<0.01; ***, p<0.001 compared to IFN-γ-stimulated control (A, B), or WT stimulated control (D, E). ns, not significant.

The binding of STAT1 to a functional IFN-γ-activated site (GAS) at −942 to −934 transactivates the expression of iNOS gene in macrophages treated with LPS and IFN-γ [29]. To investigate whether *T. congolense*-induced NO release in macrophages is also mediated via activation of iNOS GAS1 and GAS2, we transiently transfected ANA-1 and BALB.BM cells with luciferase reporter constructs carrying either wild-type (WT) or mutated GAS1, GAS2, or GAS1/2 elements in the proximal iNOS promoter sequence. ANA-1 cells transfected with WT iNOS promoter construct depicted an increase in luciferase activity over basal control in response to IFN-γ stimulation and this effect was dramatically enhanced in the presence of *T. congolense* lysate (Fig. 6A). In contrast and consistent with NO production (see Fig. 1), IFN-γ-induced iNOS gene promoter activity was significantly decreased in BALB.BM cells following *T. congolense* lysate stimulation (Fig. 6B). Both ANA-1 and BALB.BM cells transfected with iNOS-GAS1Δ displayed a significant reduction in iNOS promoter activity following stimulation with IFN-γ or IFN-γ+*T. congolense* lysate (Fig. 6D and E). Interestingly, ANA-1 cells transfected with GAS2Δ did not show a significant decrease in the iNOS promoter activity following IFN-γ or IFN-γ+*T. congolense* lysate stimulation (Fig. 6D) whereas the activity was significantly suppressed in BALB.BM cells (Fig. 6E), suggesting that GAS2-binding site is dispensable in IFN-γ/TC-induced iNOS promoter activation in ANA-1 cells while both GAS1 and GAS2 are important in BALB.BM cells. As expected, dual mutations (GAS1/2Δ) led to a clear reduction in iNOS luciferase activity in both IFN-γ alone and *T. congolense* lysate+IFN-γ-treated groups compared to respective WT iNOS-luc-transfected ANA-1 and BALB.BM cells (p<0.01, Fig. 6D, and E). Taken together, these data suggests that TC and IFN-γ induce iNOS gene expression through promoter transcriptional mechanisms. Our results also support a novel role for GAS1 in ANA-1 whereas both GAS1 and GAS2 binding sites activation in iNOS gene regulation in BALB.BM cells.

## Discussion

The aim of this study was to determine the molecular and intracellular signalling pathways that regulate nitric oxide production in macrophages following their interaction with *Trypanosoma congolense* and to see whether these vary in the relatively resistant and highly susceptible mice. Our data show that both primary as well as immortalized bone marrow-derived macrophages from the relatively resistant C57BL/6 mice produce higher amounts of NO following stimulation with IFN-γ and *T. congolense* lysate than those from the highly susceptible BALB/c mice. Although there were quantitative differences in the NO release between immortalized and primary macrophages from both C57BL/6 and BALB/c mouse strains, the overall pattern of response was similar in both cell types. Interestingly, we found that unlike ANA-1 cells, *T. congolense* lysate alone induced detectable levels of NO in BALB.BM cells. However, this effect was not observed in primary bone marrow-derived macrophages from BALB/c mice, suggesting that the immortalization processes may have impacted differently on ANA-1 and BALB.BM cell lines. Unlike immortalized cell lines, primary cell cultures more closely mimic the physiological state of cells *in vivo*. Using various techniques, we showed that MAPKs differentially regulate NO production in BALB/c and C57BL/6 macrophages in the presence of IFN-γ and *T. congolense* lysate. ERK1/2, p38, and JNK MAPK regulate both IFN-γ and *T. congolense*-induced NO release in BALB.BM macrophage cell lines, whereas only IFN-γ+ *T. congolense* signalling is affected by MAPK in ANA-1 macrophages. Interestingly, the activation of the downstream transcription factor STAT1 is indispensable for NO production in both cell lines whereas STAT3 and STAT5 are dispensable. Further analysis suggested that the binding of GAS-1 on iNOS gene promoter plays a crucial role in transcriptional activation of iNOS gene promoter in ANA-1 cells whereas both GAS1 and GAS2 were required for iNOS promoter activity in BALB.BM cell line. Collectively, our data uncovers some differential signalling pathways and enhances our understanding of the signaling messengers and transcription factors that are involved in NO release in murine macrophages following interaction with *T. congolense*.

The host protective immunity against *T. congolense* infection in mice is dependent on the production of proinflammatory mediators such as IFN-γ, TNF-α and NO [30]. Macrophages from trypanosome-infected hosts are the major source of many proinflammatory and immunoregulatory molecules, including IL-12, NO, TNF-α, IL-1 and IL-10 [31], [32], [33], [34]. Among these, NO is a pivotal effector molecule and possesses both cytostatic and cytolytic properties for the parasites [21]. In addition, we previously demonstrated that peritoneal and bone marrow-derived macrophages from C57BL/6 mice produce significantly more NO following stimulation with IFN-γ and *T. congolense* lysate than similarly treated macrophages from BALB/c mice [9]. Although these reports have clearly shown the release and importance of NO by macrophages in resistance to experimental African trypanosome infections, no study has addressed the intracellular signalling events that lead to the production of this important effector molecule, let alone the comparative differences between resistant and susceptible strains of mice. Thus, the data presented here (i) corroborate our previous findings in the pattern of NO release from macrophages of both mice strains, and (ii) provide some mechanistic details of signaling pathways involved in NO release in IFN-γ and *T. congolense*-treated macrophages. Interestingly, we found that *T. congolense* enhanced IFN-γ-induced NO release and iNOS transcriptional promoter activation in ANA-1 cells whereas it downregulated these processes in BALB.BM cells. It is conceivable that the prolonged survival in C57BL/6 mice may be attributed to this enhanced and sustained NO production compared with the BALB/c mice [9].

It has been shown earlier that IFN-γ and *T. brucei rhodesiense* sVSG initiates a cascade of p38, Erk1/2, JNK MAPK and nuclear factor kappa B (NF-κB) pathways which were suggested to eventually induce the expression of a subset of proinflammatory genes such as iNOS, TNF-α, IL-12 and IL-6 [35]. However, a definitive confirmation of the involvement of MAPK in iNOS mRNA or NO release using genetic approach or chemical inhibition was not provided. Interestingly, a convincing role of MAPK in parasite *T. cruzi-* and IFN-γ-induced NO production has been shown in J77.4 macrophages [25]. Erk1/2 and p38 MAPK were shown to play a key role in the transcriptional and post-transcriptional regulation of iNOS and TNF-α in glial cells treated with LPS in the presence or absence of IFN-γ [10]. We observed that MAPK inhibitors completely abrogated the *T. congolonse* and IFN-γ-induced NO release in BALB.BM cells. By contrast, inhibition of MAPK only affected IFN-γ signaling in ANA-1 cells, suggesting that NO release in these cells following *T. congolonse* and IFN-γ stimulation may employ additional signaling pathways, such as STATs and GAS transcription factors. Interestingly, *T. congolense* lysate alone did not exhibit a conspicuous activation of MAPK. Instead, both *T. congolense* and IFN-γ were found to exert complementary signaling events to induce NO generation. This suggests that the induction of NO production in macrophages by African trypanosomes requires a ‘priming’ effect of IFN-γ, which is consistent with our previous findings [9], [35].

Signals initiated by different microbial products or cytokine receptor engagement on immunes cells can activate STAT transcription factors leading to activation of the Janus kinases (JAKs) and proinflammatory mediators release [36]. STAT proteins are found in inactive states in cytoplasm. Once activated through cytokine receptor or microbial ligands, they dimerize, translocate to the nucleus, and regulate the expression of various genes. Activated STATs play a critical role in regulating host innate and adaptive immune responses [36]. Although STATs can activate proinflammatory mediator release independently of JAKs, this activity is completely dependent on MAPK pathways including IL-6 [11], and NO [25] release. Indeed, IFN-γ-induced activation of macrophages leads to STAT1 translocation and subsequent transcription of iNOS gene and NO release [25]. In addition, several reports suggest that IFN-γ-induced NO production in macrophages following stimulation with LPS, sVSG or other cytokines involves STAT1 phosphorylation [7], [8]. In the absence of sufficient levels of IFN-γ, exposure of macrophages to purified parasite GPI leads to inhibition of STAT1 phosphorylation and abrogation of NO production [7]. We found that pre-treatment of ANA-1 and BALB.BM cells with a STAT1-specific inhibitor, fludarabine, before *T. congolense* and IFN-γ stimulations inhibits STAT1 activation leading to abrogation of NO release. Collectively, our data and those of others suggest that STAT1 [8], [25] and GAS elements [28] are the key transcription factors that need to be activated for NO release in macrophages after *T. congolense* and IFN-γ treatment.

The GAS elements are known to bind the homodimeric form of STAT1 and previous studies show that STAT1-GAS interaction is required for the induction of iNOS gene in IFN-γ- and LPS-stimulated mouse macrophages [29]. In addition to STAT1, IFN-γ-mediated iNOS induction has also been shown to require STAT3 activation [37]. We found that stimulation with *T. congolense* enhanced IFN-γ-induced iNOS promoter activity in ANA-1 cells whereas it inhibited the iNOS transcriptional activation in BALB.BM cells. Interestingly, we found that GAS2 mutation did not significantly change iNOS promoter activity in *T. congolense* and IFN-γ-treated ANA-1 cells, suggesting that iNOS promoter activation is regulated by only GAS1. In contrast, both GAS1 and GAS2 transcription factors were required for optimal iNOS transcription in BALB.BM cells. This is the first report showing that a differential activation of GAS1 and GAS2 binding sites is required to switch ‘ON’ the iNOS gene transcription and likely NO production in both macrophage cell lines following exposure to IFN-γ and *T. congolense*.

In conclusion, our data identify the signalling pathways that are involved in NO production in macrophages from the relatively resistant and highly susceptible mice following stimulation with IFN-γ and *T. congolense* lysate. Collectively, they show that there is a coordinated but yet differential activation of MAPK, STAT1, and GAS elements for effective expression of iNOS/NO in murine macrophages. Understanding these complex signaling pathways may eventually pave the way for attractive targets conferring enhanced protection against trypanosomiasis.
